# Variants of the Ebola virus matrix protein VP40 have differential effects on oligomerization and plasma membrane interactions

**DOI:** 10.1016/j.jbc.2025.110489

**Published:** 2025-07-16

**Authors:** Balindile B. Motsa, Barsha Bhowal, Yogesh B. Narkhede, Ukesh Karki, Valentina Toro Ramirez, Samuel W. Eger, Olaf Wiest, Prem P. Chapagain, Robert V. Stahelin

**Affiliations:** 1 Borch Department of Medicinal Chemistry and Molecular Pharmacology and the Purdue Institute of Inflammation, Immunology, and Infectious Disease, Purdue University, West Lafayette, Indiana, USA; 2 Department of Chemistry and Biochemistry, University of Notre Dame, Notre Dame, Indiana, USA; 3 Department of Physics, Florida International University, Miami, Florida, USA; 4 Pharmaceutical Chemistry, Universidad CES, Medellin, Colombia; 5 Biomolecular Sciences Institute, Florida International University, Miami, Florida, USA

**Keywords:** Ebola virus, electrostatics, lipid-protein interactions, phosphatidylinositol-4,5-bisphosphate, phosphatidylserine, plasma membrane, virus assembly, virus budding, VP40

## Abstract

The Ebola virus (EBOV) has been responsible for several outbreaks in Africa over the last decade causing severe hemorrhagic fever with limited treatment options. EBOV is a lipid-enveloped filamentous negative-sense RNA virus with a genome encoding seven proteins. This includes the matrix protein VP40, which regulates assembly and budding of new virions from the inner leaflet of the host cell plasma membrane (PM). VP40 is a multifaceted protein that has different oligomeric states to regulate different parts of the virus life cycle. We investigated how two patient-derived mutations of VP40 samples (R204H and H269R) altered VP40 electrostatics, VP40 oligomeric state, interactions with PM anionic lipids and how these changes affect virus-like particle formation using both laboratory and computational approaches. The R204H mutation induced exclusive octamer formation destabilizing the dimer structure needed for proper lipid binding, PM localization, and virus-like particle formation. In contrast, H269R altered VP40 C-terminal domain (CTD) electrostatics and had similar dimer formation to WT VP40. H269R led to an increase in PM localization compared to WT VP40 and had a three-fold increase in affinity for PM anionic lipids (phosphatidylserine and PI(4,5)P_2_), consistent with the increased PM localization. Molecular dynamics simulations revealed increased interactions of H269R with phosphatidylserine by several CTD residues. These results highlight the properties of two electrostatic changes found in nature on VP40 structure and function. Understanding the effects of amino acid substitutions on VP40 biophysical properties will be helpful in explaining changes in EBOV variants that occur in nature.

The Ebola virus (EBOV) can cause a severe hemorrhagic fever with a high fatality rate. EBOV has caused several outbreaks over the last decade in Western and Sub-Saharan Africa (1) after its initial discovery in 1976 (2). EBOV is a lipid-enveloped virus with a filamentous structure and a negative strand RNA genome. EBOV has an ∼19kb genome that encodes for seven proteins including the lipid-binding matrix protein VP40 (viral protein 40 kDa) (3). VP40 has been shown to facilitate the formation and release of filamentous virus-like particles (VLPs) that are nearly indistinguishable from the real virions (4, 5, 6). These VLP systems have been used to understand the mechanisms of VP40 hijacking host proteins for protein trafficking, virus budding (7, 8, 9), as well as interactions with plasma membrane (PM) anionic lipids that facilitate formation of the matrix layer (10, 11, 12, 13). Although strides have been made in illuminating how VP40 interacts and oligomerizes into the matrix layer at the PM interface, profoundly understanding VP40 structure and function will strengthen drug targeting efforts against this protein (14, 15, 16).

VP40 can form several different structures in the virus life cycle with distinct functions (17, 18, 19). For instance, VP40 forms a dimer that is necessary for trafficking to the PM and PM lipid binding. Abrogation of the VP40 dimer leads to lack of PM localization and VLP formation (19, 20). The VP40 dimer can also oligomerize at the PM interface because of lipid binding (19, 20, 21, 22). The VP40 oligomers that form at the PM interface give rise to the virus matrix layer through VP40 dimer-dimer interactions (18, 23). VP40 also can form an RNA binding ring octamer structure through a different oligomerization interface than that used in the dimer (19, 24). The VP40 octamer does not strongly interact with anionic lipids like that of the dimer and has not been detected at the PM interface (19, 20) or in VLPs (19, 25). The VP40 octamer can also be found for WT VP40 purification albeit at a much lesser amount than the dimer (19, 26). However, the VP40 dimer stability is sensitive to experimental conditions and mutations (not only at the dimer interface), which can increase or induce exclusive octamer formation (26, 27). The VP40 octamer is thought to be necessary during infection for the transcriptional regulation of viral gene expression, which has been revealed *via* VP40 octamer use in virus minigenome systems (18, 19, 24).

Several VP40 structures have been solved revealing an N-terminal domain (NTD) and a C-terminal domain (CTD) (17, 19) (Fig. 1*A*). The NTD is critical to dimerization where the VP40 dimer is stabilized by hydrophobic and some polar interactions through an alpha-helical interface and an adjacent loop region (19). The NTD is also critical for VP40 ring octamer stability and RNA binding (19, 24). The VP40 CTD is the primary site of anionic PM lipid interactions (13, 19, 20) (Fig. 1*A*). VP40 associates with PM lipids predominantly through electrostatic interactions and H-bonding with phosphatidylserine (PS) and phosphatidylinositol 4,5-bisphosphate (PI(4,5)P_2_). There are two C-terminal regions rich in cationic residues that have been shown to mediate interactions with PS and PI(4,5)P_2_ (12, 13, 20, 22). These C-terminal regions have been referred to as motifs CTD-1 and CTD-2, where CTD-1 includes a cationic loop spanning Lys^221^ to Ser^228^ and CTD-2 encompasses several cationic residues from His^269^ to Lys^279^.

**Figure 1:**
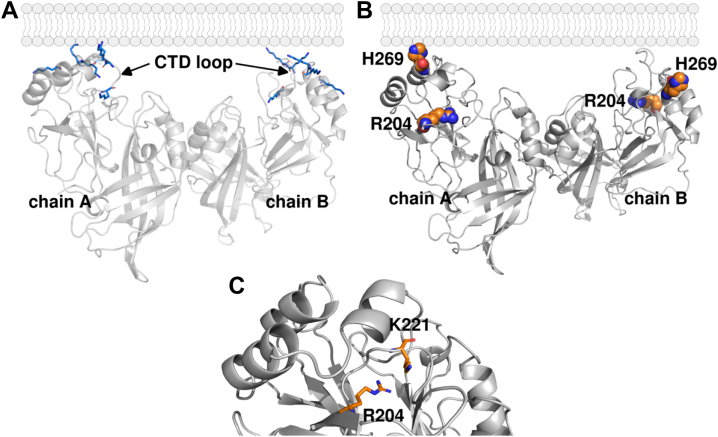
**Ebola virus VP40 dimer structure highlighting the Arg^204^ and His^269^ residues and orientation at the membrane interface.***A*, the VP40 dimer structure is shown (PDB 4LDB) positioned based on previously characterized binding orientations (13, 20, 28). The CTD-1 and CTD-2 cationic patch lysine residues are depicted with *blue sticks*. *B*, position of Arg^204^ and His^269^ (depicted as *spheres*) in both chains of the VP40 dimer. *C*, positioning of Arg^204^ with respect to Lys^221^ (13, 20) in PDB 4LDB. CTD, C-terminal domain; PDB, Protein Data Bank.

Electrostatic changes on the VP40 CTD surface have been shown to reduce or inhibit lipid binding when Lys residues in CTD-1 or CTD-2 were changed to Ala or Glu (28). The reduced lipid binding of these VP40 mutations leads to a reduction in PM localization and little to no VLP formation (28). In contrast, it has been shown that increasing the cationic charge by +1 in the CTD can increase VP40 lipid binding affinity and PM localization of VP40 (28). Thus, VP40 is sensitive to singular slight electrostatic change on the CTD surface in or near CTD-1 or CTD-2. VP40 PM interactions trigger VP40 oligomerization, initially by PS interactions (11, 12, 29) as well as PI(4,5)P_2_ interactions, the latter of which stabilizes VP40 oligomers that form (13, 22). VP40 oligomerization *via* dimer-dimer interactions is critical to matrix layer formation and effective VLP and virion formation (19, 23). The VP40 dimer-dimer interactions are formed *via* CTD-CTD interactions between each dimer through a hydrophobic interface.

VP40 is considered a target for small molecule direct acting antivirals (14, 15, 16) and understanding mechanisms by which VP40 structure can be altered is critical to this venture (26, 27, 30). Scores of VP40 mutations have been found *via* sequencing of patient samples from several previous outbreaks (31, 32, 33). The Nextstrain database (www.nextstrain.org) lists genomic epidemiology of several previous EBOV outbreak samples with sequence changes between the known roots of each outbreak. Scrutinizing individual VP40 mutations detected in different patient samples is critical in understanding how VP40 structure and function may be altered. Recent studies have detailed mechanisms by which electrostatic changes in the VP40 CTD alters interactions with PS and PI(4,5)P_2_ (13, 28) and that VP40 interactions with these lipids are driven by cationic residues on the surface of the CTD (13, 20). Two mutations detected from patient samples indicated charge changes that altered CTD electrostatics including R204H and H269R. Thus, we examined in detail *in vitro*, in cells, and *in silico*, how R204H and H269R influenced VP40 oligomerization state, PM interactions, and formation of VLPs.

## Results

### VP40 Arg^204^ and His^269^ site saturation analysis

The 2013 to 2016 EBOV outbreak was the largest and most deadly in history with over 28,000 cases and 11,000 deaths (32). Deep sequencing of patient samples from this outbreak allowed for discovery of multiple lineages of virus as it passed through humans in Guinea, Liberia, and Sierra Leone (33, 34, 35). This deep sequencing led to discovery of at least 40 mutations in VP40 in different lineages and individual patients ((36), www.nextstrain.org). Deep sequencing has also been performed on EBOV samples in the Democratic Republic of Congo (37) and Sudan virus samples in Uganda (38). Deep sequencing analysis has revealed mutations of VP40 in His^269^, which is at the membrane binding interface (Fig. 1*B*) and Arg^204^, which resides at the interface of the VP40 CTD and NTD (Fig. 1*C*). Despite detection of these mutations *via* sequencing, the effects of mutation on disease severity or patient outcomes are not known.

**Figure 2:**
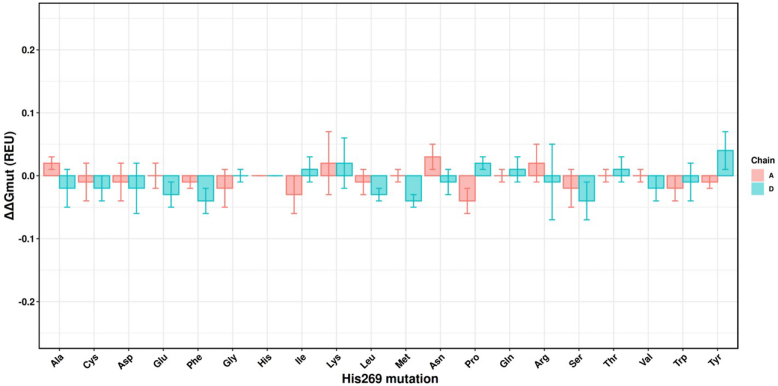
**Site saturation mutagenesis for His^269^ of WT VP40.** Reported values and error bars have been averaged over 100 models that represent 100 cycles of mutagenesis. Positive values of predicted ΔG imply destabilization of dimer while negative ΔG values imply increased stabilization of the dimer. Mutation of His269 to lysine or arginine can be clearly seen as destabilizing for dimer.

Computational site saturation mutagenesis (SSM) was previously used to identify Arg^204^ as a CTD residue critical to dimer stability. Mutating Arg^204^ to the other 19 amino acids was for the most part favorable in prediction of dimer disruption ((27), Fig. S1). Cellular experiments demonstrated R204A, R204I, and R204P greatly reduced PM localization of VP40 strongly suggesting VP40 dimerization impairment (27). Next, we performed SSM on His^269^ to assess the effect of residue substitution on this position in the CTD cationic patch (CTD-2). SSM (Fig. 2) demonstrated similar free energy changes among all 19 amino acid substitutions strongly suggesting H269R would not favorably disrupt the VP40 dimer interface. In a typical use scenario, SSM performed with Rosetta-based flex ddG yields one of three values–positive (>0 Rosetta energy units (REU)), neutral (=0 REU), and negative (<0 REU). A positive value post mutation is predicted to destabilize a protein-protein interaction (PPI), which in the current work manifests as decreased dimer stability. On the contrary, a negative value post mutation would mean favorable conditions for PPIs and thereby increased dimer stability. A neutral value is predicted to have no effect on PPIs/dimer stability with respect to WT protein. This fact has been observed in numerous studies (39, 40, 41).

**Figure 3:**
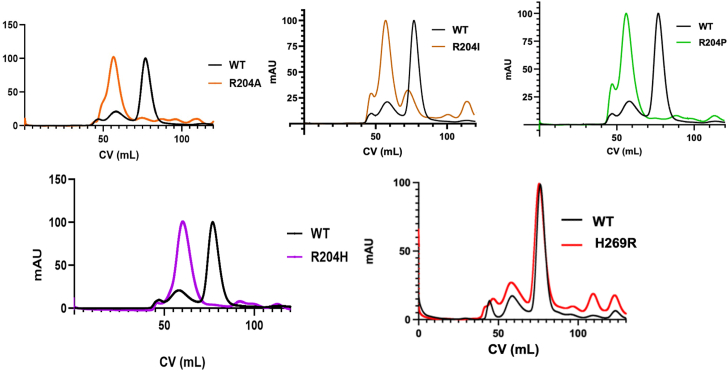
**VP40 dimer and octamer assessment from *in vitro* purification.** Size-exclusion chromatography was used to assess VP40 octamer and dimer formation for WT VP40, R204A, R204I, R204P, R204H, and H269R. All Arg^204^ mutants displayed an increased propensity toward the octamer form compared to WT VP40 and very little to no dimer present. In some of the plots, the same WT VP40 profile is used for the overlay.

To test the effects of mutation on Arg^204^ or His^269^ on VP40 dimer formation, we expressed and purified each mutation and used size-exclusion chromatography to assess dimer and octamer formation of each construct (26, 27). For the case of Arg^204^ we prepared the R204H mutant detected in 2019 in the Democratic Republic of Congo (BTB17389) (www.nextstrain.org). It should be noted that R204H has only been detected along with G29S in VP40; however, we chose to simplify the study and focus on Arg^204^ in the CTD for two reasons. First, the secondary structure of first 43 amino acids of VP40 is unknown (17, 19) and second, the first 43 amino acids do not appreciably contribute to lipid binding or dimer formation of VP40 (19, 20). We also prepared three additional Arg^204^ mutations (R204A, R204I, and R204P) for dimer assessment because these mutations were previously predicted to be destabilizing but only monitored for PM localization in cell studies (26). For His^269^, we prepared H269R to assess this mutant as previously detected from a sample in 2014 in Guinea (EM_076615) (36). In line with our previous predictions, R204A, R204I, and R204P destabilized the VP40 dimer leading mainly to detection of VP40 octamers (Fig. 3). Similarly, R204H also precluded dimer formation and led to formation of a VP40 octamer signifying the critical Arg at this position (Fig. 3). Arg^204^ is part of the CTD cationic patch (Fig. 2, *B* and *C*) yet critical to dimer stability as evidenced by our previous computational and cellular studies as well as *in vitro* studies presented herein. H269R behaved like WT VP40 with a predominant dimer peak and much smaller octamer peak (Fig. 3). This is also in line with SSM where mutations of His^269^ did not significantly alter the energetics of dimer stability (Fig. 2).

### VP40 H269R increases PM localization and membrane binding ability

**Figure 4:**
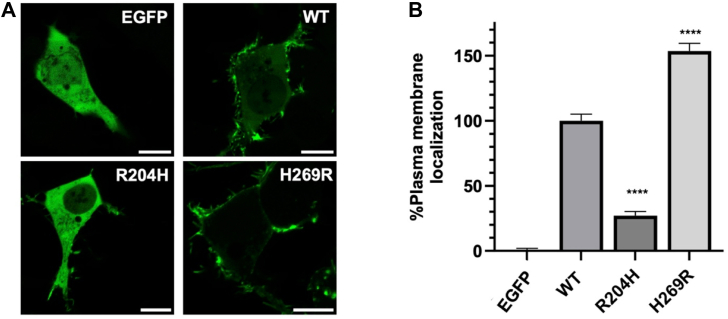
**Cellular localization of WT VP40 and respective mutants in HEK293 cells.***A*, representative images of EGFP, EGFP-WT-VP40, EGFP-VP40-R204H, and EGFP-VP40-H269R constructs imaged 24 h post transfection. The scale bar represents 10 μm. *B*, average PM localization for each construct. Image analysis was done in Image J to determine percent PM localization (%PM localization) for each construct. %PM localization was defined as (PM intensity/(PM intensity + cytosol intensity))∗100. Data were normalized to %PM localization of WT as 100% localization and %PM localization of EGFP as 0% localization. A WGA-Alex647 PM marker was used to mark the PM in each cell. Error bars are the SEM from three independent experiments with at least 10 images analyzed per replicate. A one-way ANOVA was performed with multiple comparisons compared with WT PM localization (∗∗∗∗*p* < 0.0001). EGFP, enhanced green fluorescent protein; PM, plasma membrane

VP40 assembles at the inner leaflet of the PM where VP40 oligomerization occurs in response to lipid binding (10, 11, 21), facilitating assembly of the matrix layer that gives rise to virions and VLPs. Therefore, PM localization has often been a readout for effects of mutation on VP40 lipid binding and relative affinity for PM lipids (20, 22, 26). Confocal imaging was performed to quantify PM localization of EGFP-WT-VP40, R204H, H269R, and enhanced green fluorescent protein (EGFP) as a negative control (Fig. 4). HEK293 cells were imaged 24 h post transfection for three independent experiments for each construct (Fig. 4). WT VP40 exhibited robust PM localization and pre-VLP formation as evidenced by green filamentous protrusions as previously described (13, 22, 26). In contrast, EGFP displayed an overall diffuse localization. The R204H mutation also showed predominant cytoplasmic localization, with little to no evidence of PM assembly. Notably, the H269R mutant had prominent localization at the PM, with minimal cytosolic GFP signal (Fig. 4). Quantification of PM localization, which utilized PM labeling with WGA-Alexa647 as done in several previous studies (12, 26), demonstrated a significant increase in H269R PM localization compared to WT VP40 and a significant decrease in R204H PM localization, which was in line with visual scrutiny of the confocal images (Fig. 4).

**Figure 5:**
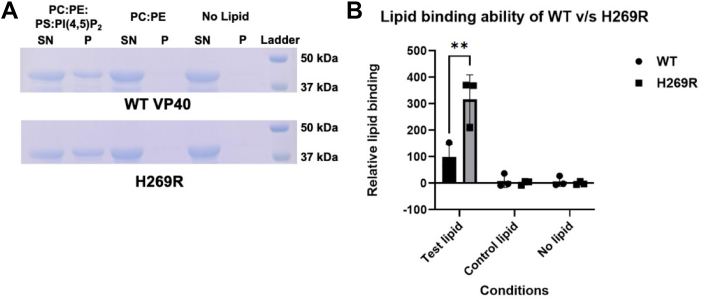
**Lipid binding analysis of WT VP40 and H269R.** Lipid vesicles were prepared (control membrane: POPC:POPE (80:20) or test membrane: POPC:POPE:POPS:PI(4,5)P_2_ (55:20:20:5)) and incubated with the respective VP40 protein. The presence of a protein band in the pellet fraction (P) indicates binding to the lipid vesicle. *A*, representative SDS-PAGE gels showing supernatant (SN) and pellet (P) fractions after the lipid vesicles were incubated with WT and H269R VP40 dimers and centrifuged to separate the lipid bound and unbound fraction. H269R shows increased binding to the POPS and PI(4,5)P_2_ containing membrane compared to the WT. Both WT and the mutant do not bind to the control membrane. *B*, ImageJ was used to quantify the band intensity for each sample, n = 3 all samples. Relative lipid binding indicates the ratio of pellet band intensity to the total intensity of pellet and supernatant bands combined and normalized to WT as 1.0. Error bars are the standard deviation. A two-way ANOVA with multiple comparison to WT was performed (∗∗*p* < 0.01). PI(4,5)P_2_, phosphatidylinositol-(4,5)-bisphosphate; POPC, 1-palmitoryl-2-oleoyl-glycero-3-phosphocholine; POPE, 1-palmitoyl-2-oleoyl-sn-glycero-3-phosphoethanolamine; POPS, 1-palmitoyl-2-oleoyl-sn-glycero-3-phospho-L-serine.

To evaluate lipid binding of H269R compared to WT VP40 we prepared multilamellar lipid vesicles (MLVs), which have been previously used to assess VP40 lipid binding in a centrifugation assay (22). R204H was excluded from these experiments since it led to exclusive octamer formation, octamers have greatly reduced lipid binding (20) and are not relevant to PM localization and budding of VP40 (19). A control membrane of 1-palmitoryl-2-oleoyl-glycero-3-phosphocholine/1-palmitoyl-2-oleoyl-sn-glycero-3-phosphoethanolamine (POPC/POPE) (80:20) and active membrane of POPC/POPE/POPS:PI(4,5)P2 (55:20:20:5) was used, VP40 has been shown to selectively bind these anionic lipids at the PM (11, 12, 13, 22). SDS-PAGE was used to visualize the VP40 protein band in the supernatant or pellet fraction of each MLV sample or a control assay of no lipid present, which was used to ensure VP40 was not pelleting in the absence of lipid vesicles (Fig. 5*A*). As shown in Figure 5*A*, neither WT VP40 or H269R was detected following centrifugation in the pellet fraction when no lipid was present or when POPC:POPE vesicles were used. Furthermore, WT VP40 associated with vesicles containing PS and PI(4,5)P_2_, and visually H269R appeared to have an increase in the VP40 amount in the pellet fraction and a decrease in VP40 in the supernatant fraction (Fig. 5*A*). Experiments were performed to quantify the VP40 band intensity in each sample (Fig. 5*B*). As expected, negligible binding was quantified for either protein to POPC:POPE and there was no detectable protein in the pellet fraction of the no lipid samples. In contrast, both WT VP40 and H269R displayed robust binding to vesicles containing PS and PI(4,5)P_2_ where H269R had a 3-fold statistically significant increase in binding to these anionic PM lipids compared to WT VP40 (Fig. 5*B*).

### VP40 R204H abrogates VLP budding

**Figure 6:**
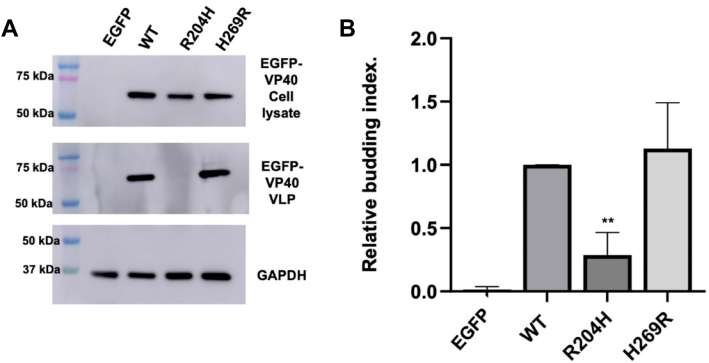
**VLP analysis of WT VP40 and respective mutations expressed in HEK293 cells.** HEK293 cells were transfected with plasmid DNA encoding EGFP, EGFP-WT-VP40, EGFP-VP40-R204H, or EGFP-VP40-H269R. *A*, representative blots of WT VP40 and respective mutations in the VLP or cell lysate fractions. The VLPs and cell lysates were collected 48 h post transfection and subjected to Western blot using anti-GFP. GAPDH was used as a loading control for these experiments. Molecular weight markers are shown for all blots to verify band size for EGFP-VP40 and GAPDH. *B*, ImageJ was used to quantify the band intensity for each sample (n = 3 for all samples). VLP budding efficiency was quantified and normalized to WT as 1.0. This quantification was used to determine the budding index (*y*-axis) for each mutation, which is a relative change in VLP formation compared to WT VP40. Error bars are the standard deviation; A one-way ANOVA with multiple comparison to WT was performed (∗∗*p* < 0.01). EGFP, enhanced green fluorescent protein; VLP, virus-like particle.

We next assessed VLP formation of R204H and H269R compared to WT VP40 to determine if changes in dimerization, lipid binding or PM localization altered VLP formation (Fig. 6). VLP budding assays have been a common way of comparing budding efficiency of WT VP40 and respective mutations (13, 28, 42). VLPs were purified from the supernatant of HEK293 cells expressing EGFP-WT-VP40, EGFP-R204H, or EGFP-H269R 48 h after plasmid transfection. Following VLP purification, cell lysate expression and VLP density of VP40 were assessed using Western blot (Fig. 6*A*). Cellular levels of GAPDH were also evaluated to compare total cell background for each condition to allow robust comparison among samples. R204H led to significant decrease in VLP formation with VLPs barely detectable *via* VP40 band density analysis (Fig. 6, *A* and *B*). This is consistent with R204H forming exclusively an octamer *in vitro* and lack of PM localization in HEK293 cells. H269R had similar VLP formation to WT VP40 indicating the increased PM localization and membrane affinity was not sufficient to increase VLP production from cells (Fig. 6*B*).

### Molecular dynamic simulations of VP40, R204H, and H269R

**Figure 7:**
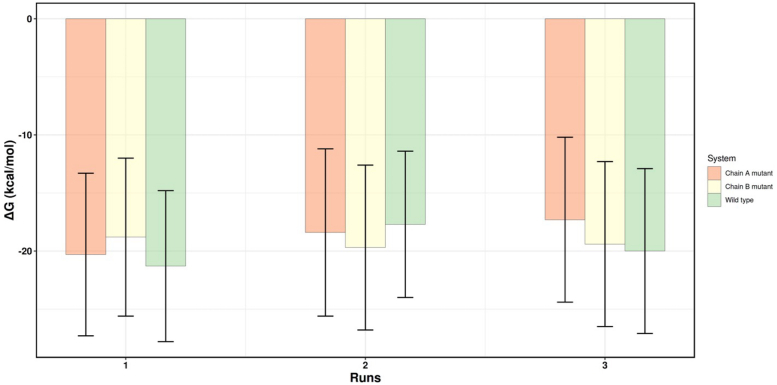
**Relative binding free energy calculations for WT VP40, chain A R204H and chain B R204H mutants.** Each individual bar’s value and its corresponding standard deviation has been calculated over an ensemble of 1000 frames corresponding to 100 ns of simulation under constant pressure and temperature. The individual chain mutants refer to the chain where the mutation was carried out with the other chain corresponding to WT VP40 sequence.

**Figure 8:**
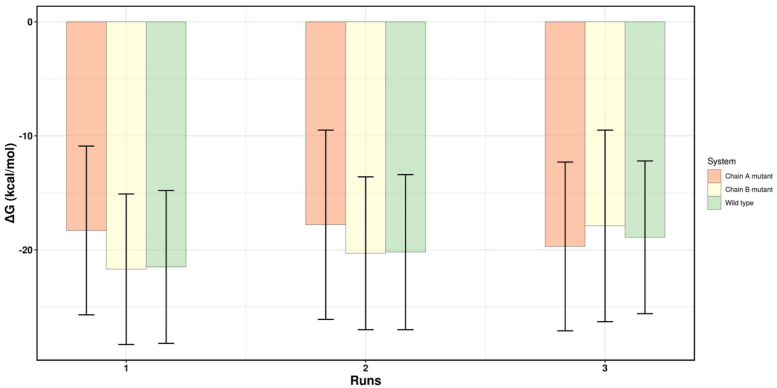
**Relative binding free energy calculations for WT VP40, chain A H269R and chain B H269R mutants.** Each individual bar’s value and its corresponding standard deviation has been calculated over an ensemble of 1000 frames corresponding to 100 ns of simulation under constant pressure and temperature. The individual chain mutants refer to the chain where the mutation was carried out with the other chain corresponding to WT sequence.

**Figure 9:**
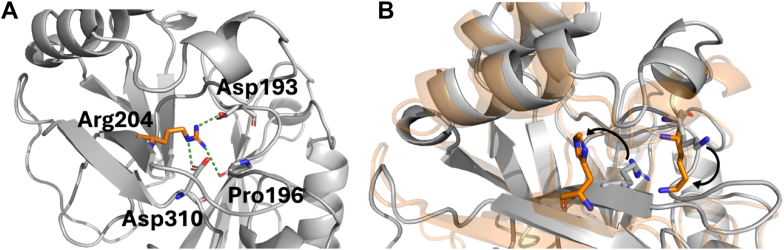
**Hydrogen bonding of Arg^204^ and dynamic effects of R204H mutation.***A*, The VP40 dimer is shown with hydrogen bonds being formed by the guanidium side chain of Arg^204^ (PDB 4LDB) *B*, R204H mutation leads to the histidine side chain flipping out (*orange sticks*) accompanied by inward rotation of Lys^221^ side chain (*orange sticks*). The *gray* cartoon structure represents the crystal structure, while the *orange* transparent one is averaged from MD simulations. MD, molecular dynamics.

**Figure 10:**
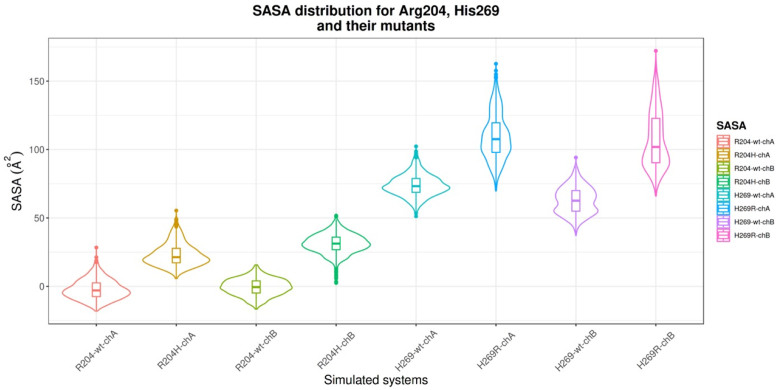
**Solvent accessible surface area (SASA) distributions for WT Arg^204^, His^269^, R204H, and H269R mutants.** Each violin plot represents the SASA distribution averaged over triplicate 100 ns MD simulations. Both R204H and H269R mutations reflect an increased exposure to solvent as evident in upward shift of SASA for the mutants irrespective of the chain where the mutation was carried out. MD, molecular dynamics.

**Figure 11:**
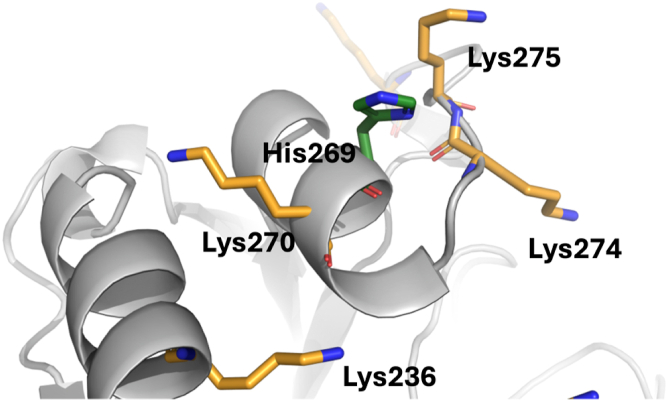
**Positioning of His^269^ in VP40.** His^269^ (*green sticks*) is nestled among lipid binding residues Lys^270^, Lys^274^, and Lys^275^ (*orange sticks*) that are key constituents of VP40 cationic patch facing the solvent or inner leaflet of the plasma membrane.

**Figure 12:**
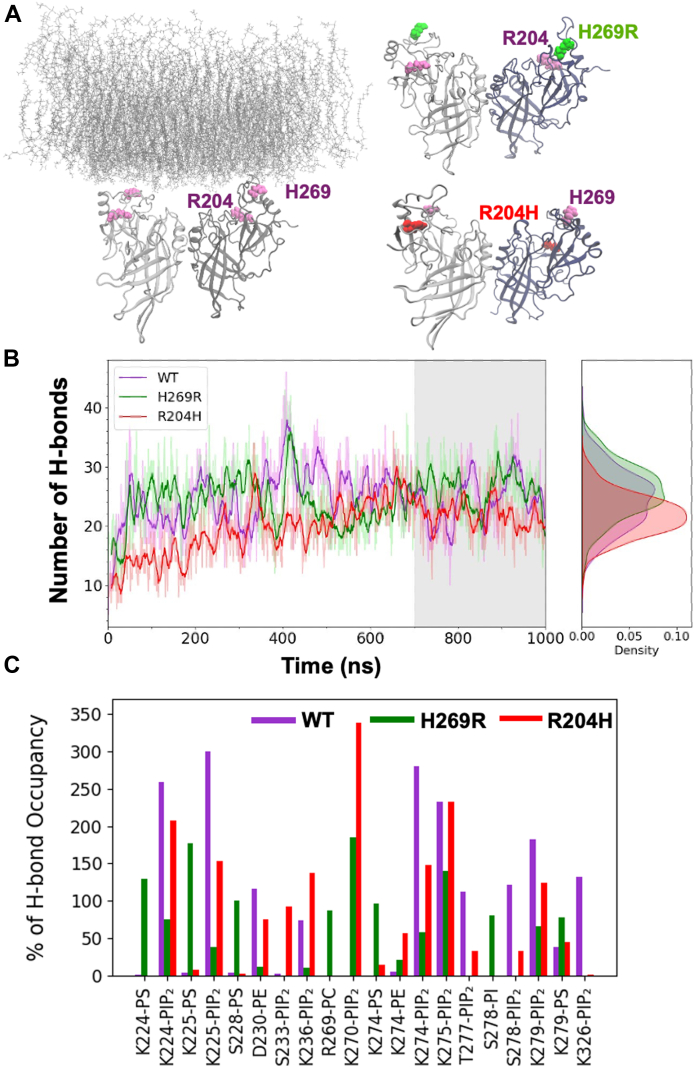
**Molecular dynamics simulations of WT VP40, R204H, and H269R with a plasma membrane mimetic bilayer.***A*, MD simulations were performed with WT VP40, R204H, and H269R for 1000 ns with a PM mimetic. *B*, the number of hydrogen bonds made with the plasma membrane model for WT VP40, R204H, and H269R are shown through the MD simulation time. The *right* panel displays vertical kernel density estimates (KDEs) of hydrogen bond counts with the final 300 ns segment, illustrating distributional behavior of each variant-lipid hydrogen bond. *C*, percent of hydrogen bond occupancy plotted for the portion of the VP40 CTD that interacts with the plasma membrane, including CTD loops 1 and 2 for WT VP40, R204H, and H269R. CTD, C-terminal domain; MD, molecular dynamics; PM, plasma membrane.

We performed molecular dynamics (MD) simulations of WT VP40, as well as R204H and H269R in free state and in association with a model membrane to determine how the changes in these residues affect dimerization and membrane binding (Figure 7, Figure 8, Figure 9, Figure 10, Figure 11, Figure 12; Tables S1 and S2). The relative binding free energy analysis carried out using the molecular mechanics Poisson-Boltzmann surface area (MM-PBSA) method (43), revealed that a R204H mutation in chain A was more destabilizing for the dimer (2.7 kcal/mol) as compared to chain B (0.6 kcal/mol) (Fig. 7 and Table S1) over triplicate 100 ns MD simulations. The H269R mutation was predicted to result in similar dimer destabilization across either chain (1.7 kcal/mol for chain A *versus* 1.1 kcal/mol for chain B; Fig. 8 and Table S2). Structural inspections of the crystal structure of VP40 reveal that Arg^204^ is buried in the cationic patch-CTD region and its mutation to histidine is accompanied by disruption of stabilizing interactions in between Arg^204^ and Asp^193^, Pro^196^, and Asp^310^ (Fig. 9*A*). Mutation of these residues instead of Arg^204^ can also alter the position and orientation of Arg^204^ that may modulate its interactions with its neighboring residues as well as the PM resulting in dimer destabilization or decreased PM affinity. Moreover, the guanidium side chain of R204 assists in pointing the charged δ-Nitrogen of Lys221 toward the exterior of protein surface, maximizing its interaction with the inner leaflet of PM. MD simulations show that R204H mutation is accompanied with the outward flip of the histidine side chain along with a simultaneous inward rotation of Lys^221^ (Fig. 9*B*). Solvent accessible surface area (SASA) analysis of native VP40, R204H, and H269R mutants revealed an increase in the total SASA of the mutants relative to WT VP40. The results were unaffected irrespective of the chain the mutation occurs in and is reproducible across the triplicate simulations (Figs. 10, S2 and S3).

The SASA distribution for R204H mutant in respect to VP40 implies a gradual conformational change upon mutation as shown by a narrow distribution with a single peak. Contrary to Arg^204^, His^269^ is more solvent exposed and sandwiched in between Lys^270^ on one side and Lys^274-275^ on the other (Fig. 11). The His^269^ side chain does not have notable hydrogen bonding interaction that might contribute to conformational stabilization in its vicinity. Like the R204H mutation, the H269R mutation is accompanied with an increase in SASA compared to VP40 irrespective of the site of mutation across all simulations (Figs. 10 and S3). The H269R mutation is also accompanied with conformational changes that manifest as a broad distribution of the SASA along with two instances of noticeable changes in the measured SASA. The conformational changes are clearly visible when the RMSD for R204H and H269R mutations across both chains are compared to WT VP40 simulations (Fig. S4). Moreover, the comparative increased SASA values for H269R relative to VP40 and R204H mutant can be attributed to its location and increased solvent exposure from the outset of the simulation.

Since R204H could not be isolated as a dimer *in vitro*, the MD simulations serve as a model to predict how R204H may impact VP40 dimer membrane interactions since some VP40 mutations seem more resistant to strict oligomer formation in cells (26). An established bilayer system was used that recapitulates the asymmetric nature of the PM (13, 28) for a simulation time of 1000 ns (Fig. 12*A*). The WT VP40 associated with the lipid bilayer in the first 50 ns of the simulation time (Fig. 12*B*) and maximized interactions at ∼400 ns as assess by VP40 dimer H-bond formation with the membrane. For WT VP40, this maximal interaction was followed by a relatively stable membrane binding interaction from 450 ns to the end of the simulation time. In contrast, R204H associated with the membrane much more slowly than WT over the first 350 ns and did not reach the maximum membrane interaction until ∼650 ns simulation time. The R204H membrane interaction was then relatively stable until the end of the simulation. H269R was similar to WT in that it quickly associated with the membrane increasing H-bond interactions early in the simulation time and reached a maximum of binding at ∼400 ns. H269R then had a small dip in interactions from ∼450 to 550 ns and the membrane binding then stabilized from 600 ns to the end of the simulation time in a similar fashion to WT VP40.

Hydrogen bond occupancy was then elucidated for WT, R204H, and H269R for two cationic loop regions (CTD-1 and CTD-2) that have been shown to robustly interact with PS and PI(4,5)P_2_ in previous studies (13, 20, 28). CTD-1 encompasses Lys^221^-Ser^233^ while CTD-2 includes Arg^269^-Lys^279^. The percent of H-bond occupancy for CTD-1 and CTD-2, as well as several residues outside these regions is shown in Figure 12*C*. WT VP40 displayed interactions of Lys^224^, Lys^225^, Lys^236^, Lys^274^, Lys^275^, Thr^277^, Ser^278^, Lys^279^, and Lys^326^ with PI(4,5)P_2_. In contrast, R204H had fewer interactions with PI(4,5)P_2_ for Lys^224^, Lys^225^, Lys^274^, Thr^277^, Ser^278^, Lys^279^, and Lys^326^. Despite overall fewer membrane interactions for R204H compared to WT, some residues in R204H did interact more prominently with the membrane, this included the following: Ser^233^, Lys^236^, and Lys^270^ with PI(4,5)P_2_. H269R had several residue interactions increase H-bond occupancy when compared to WT. This included: Lys^224^, Lys^225^, Ser^228^, Lys^274^, and Lys^279^ with PS. Notably, H269R also had increased interactions of Lys^270^ with PI(4,5)P_2_, Ser^278^ with phosphatidylinositol (PI) and Arg^269^ (the introduced residue) with phosphatdiylcholine (PC). Despite these increases in H-bonding for H269R, H269R did have some H-bond reductions with PI(4,5)P_2_ for several residues including Lys^224^, Lys^225^, Lys^236^, Lys^274^, Lys^275^, Lys^279^, and Lys^326^. The increased membrane affinity of H269R in *in vitro* binding and PM localization measurements is consistent with H269R increasing interactions with PS and PI(4,5)P_2_ in the MD simulations where increased binding to PS, PC, and PI was observed. Whereas the lack of PM localization of R204H in cell studies is consistent with MD simulations displaying a reduction in membrane binding for any R204H dimers that may form *in cellulo*.

## Discussion

To the best of our knowledge, this is the first study that examined EBOV VP40 mutations identified in patient samples. This work extends the critical role of electrostatic interactions at lipid-protein interaction interfaces between VP40 and the host cell PM inner leaflet (11, 12, 13, 20, 23, 28). Although more than 40 mutations of VP40 are known in the Nextstrain database, we chose to first examine R204H (2019 Democratic Republic of Congo BTB17389) and H269R (2014 Guinea EM_076615) as they make electrostatic changes on the VP40 lipid binding interface. Further, they are located within or adjacent to cationic amino acids that interact with PM anionic lipids (13, 20) and an increase of +1 charge to VP40 can increase anionic lipid binding and VLP formation (28).

The study integrates computational assessment of mutagenesis of the Arg^204^ and His^269^ positions to assess impacts on dimer stability with *in vitro* and cellular studies on select mutations. Mutagenesis was performed to evaluate how changing the electrostatics of Arg^204^ and His^269^ at the lipid binding interface affects different stages of the viral lifecycle. R204H served as a disruptor of dimer formation whereas H269R formed dimers like WT VP40. Cellular imaging demonstrated different effects of R204H and H269R on PM localization. R204H displayed a significant reduction in PM localization whereas H269R increased PM localization. Lipid binding assessment indicated a 3-fold increase in H269R association with PS and PI(4,5)P_2_, which was supported by MD simulations of H269R at the membrane interface and increased contacts with PS and PI(4,5)P_2_. Since R204H formed nearly exclusive octamers (Fig. 3), lipid binding of R204H octamers was not examined as the octamer has a sharp decrease in anionic lipid binding (20) and octamers have not been detected in PM interactions or VLP formation. Despite the increased PM affinity, H269R did not significantly increase VLP formation although a trend toward an increase was detected. In support of R204H destabilizing the VP40 dimer structure, R204H led to a marked reduction in VLP formation.

VP40 dimers are critical for proper trafficking to the PM for interaction with anionic lipids and VP40 oligomerization through CTD-CTD interactions for assembly and budding of VLPs (13, 19, 20, 23, 28). VP40 can also form an RNA-binding octameric ring that is thought to regulate virus transcription (18, 19, 24). Detailed structural and mechanistic studies have demonstrated that disrupting dimer formation can inhibit VP40 assembly and budding (19, 20, 28). Notably, mutations that alter dimer stability that are not part of the anionic lipid binding interface can shift VP40 equilibrium toward the octameric ring (19, 20, 26, 27, 44). Our assessment of the Arg^204^ position indicates it is a critical position and residue for dimer stability. Site saturation measurements previously published (27) and examined herein (Fig. S1) demonstrate mutation of Arg^204^ to other amino acids such as Ala, Ile, Pro, and His disrupt dimer formation favoring robust octamer stability (Fig. 3). MD simulations indicate Arg^204^ forms important stabilizing interactions with Asp^193^, Pro^196^, and Asp^310^ (Fig. 9) where R204H disrupts these stabilizing interactions and causes an inward turn of CTD-1 Lys^221^. Cellular experiments confirm these simulations as R204H lacks PM localization despite MD simulation predictions (Fig. 12) that R204H would only slightly decrease lipid interactions. Thus, it is most likely that R204H forms an octamer in cells akin to what we detected with *in vitro* measurements. In contrast, H269R behaved like WT VP40 with respect to robust dimer formation *in vitro* as predicted by site saturation measurements. The stability of dimer formation for H269R *versus* R204H is further supported by the solvent accessibility of His^269^ (Fig. 11) compared to Arg^204^ (Fig. 9) as Arg^204^ makes critical contacts with other amino acids.

A hall mark of VP40 PM assembly and budding is selective interactions with PS and PI(4,5)P_2_ at the PM inner leaflet. The VP40 dimer interacts with PM anionic lipids with several Lys residues located in CTD-1 and CTD-2 (13, 19, 20). The increased lipid binding by introduction of a +1 charge (H269R) is in line with a recent study that demonstrated introduction of Arg residues at positions 198 and 201 in a 198-GNSG-201 loop region increased PM localization and PS binding affinity (28). In contrast, an Asp at position 198 decreased affinity for PS containing vesicles and reduced PM localization. His^269^ lies between several other important Lys residues in CTD-2 (Lys^270^, Lys^274^, and Lys^275^) that interact with the PM and PI(4,5)P_2_ specifically (13). Thus, introduction of an Arg to this region demonstrates an increase in cationic charge near CTD-2, which is sufficient to increase PM binding affinity of VP40. MD simulations suggested that H269R made more contacts with PM lipids and will form more H-bonds with the PM model membrane than WT VP40. These MD simulations are supported by the 3-fold increase in affinity of H269R for PS and PI(4,5)P_2_ containing membranes. The critical nature of electrostatic changes on or near the VP40 membrane binding interface has been examined in previous studies (13, 20, 28). In many cases, the mutation of a single Lys residue to Ala greatly impairs PM localization and VLP formation through a reduction of PS and/or PI(4,5)P_2_ binding affinity (13). In some cases, an increase in PM affinity through a single cationic charge increment led to more significant VLP formation (28) whereas in the case of H269R a nonstatistically significant increase in VLP formation was detected. A limitation of the current study and the field as a whole is that the dynamics and conformational changes of VP40 that occurs at the PM interface that regulate VLP formation are not well understood. In addition, changes in VP40 or VP40 interactions with the PM that occur at the scission step of budding have not been elucidated. It is possible that different regions of VP40 (*e.g.*, CTD-1 and CTD-2) play important roles of engagement at different steps of assembly. Thus, a detailed mechanistic investigation of VP40-dependent budding is still needed and will require different snapshots of VP40 structure and host interactions at the different stages of assembly and egress.

Overall, this work demonstrates how single mutations that have been detected in patient samples can have changes on VP40 structure and function. Mutation of Arg^204^ to His and other amino acids (Fig. 3) leads predominantly to VP40 octamer formation *in vitro* and precludes PM localization (Fig. 4, *A* and *B* and (26)). H269R formed stable dimers and increased PM localization *via* a 3-fold increase in binding to PS and PI(4,5)P_2_. Despite this increase in PM interactions, H269R VLP production increases were not statistically significant although a trend toward an increase was detected. The consequences of R204H and H269R in nature are not known or correlated with patient sample EBOV genome sequencing. From these studies it would be predicted that R204H would slow assembly, budding, and spread whereas H269R would lead to more efficient PM interactions but not necessarily an increase in virus budding. The characterization of the two mutations herein is just the first step in understanding the effects of VP40 mutations that have been detected in nature. Several other electrostatic altering mutations have been detected in Ebola virus VP40 (www.nextstrain.org) including G223R (2018-2021 Ebola epidemic BEN42629), G226R (2013-2016 West African Ebola epidemic LIBR10224), Q245R (2013-2016 West African Ebola epidemic EM_COY_2015_016267), and D312G (2018-2021 Ebola epidemic BTB238). These electrostatic changes are all in the CTD and in the case of G223R and G226R in CTD-1. Since these residues are adjacent to key Lys residues previously shown to mediate lipid interactions (13, 20), they may further promote electrostatic interactions with the anionic membrane and clustering of PS and PI(4,5)P_2_ (11, 12, 13, 20, 28). We hypothesize that these four electrostatic changes will increase affinity for the PM anionic lipids but how these mutations contribute to VP40 structure and function properties will require integrated *in vitro*, computational, and cellular studies to test if they alter VP40 dimer stability or VLP formation. Nonetheless, the current study provides a template to examine VP40 mutations found in nature to reveal structural and functional consequences.

## Experimental procedures

### Plasmids, protein expression, and purification

The mammalian expression plasmid for VP40 was created by subcloning VP40 into EGFP-pcDNA3.1 as previously described (21). EGFP-VP40 mutants in pcDNA3.1 were prepared and purchased from Gene Universal. WT His_6_-VP40 and mutants were grown and purified as previously described (28). Following Ni-NTA affinity chromatography elution, WT VP40 and VP40 mutants were further purified using size exclusion column on a Hiload 16/600 Superdex 200 pg column (Cytiva Life Sciences). The desired fractions were collected, concentrated, and stored in 10 mM Hepes, pH 8.0, containing 150 mM NaCl. A sample of the purified protein was run on an SDS-PAGE gel and stained with Coomassie Brilliant Blue to confirm the appearance of a strong band at ∼40 kDa. Protein concentrations were determined using the Pierce BCA Protein Assay (Thermo Fisher Scientific) and the protein was stored at 4 °C for no longer than 2 weeks.

### Site saturation analysis

Using a previously published protocol (27), site saturation mutagenesis was performed in Rosetta (44) on a homology model of WT VP40 (Protein Data Bank, PDB 4LDB) at various positions of the NTD-NTD interface, a 5 Å region around the cationic patch and of CTD-CTD interface in each chain of the VP40 homodimer. The resultant free energy changes for mutations resulted from an ensemble of 100 models, with each model resulting from 50,000 steps of conformational diversity generation using the backrub method and equal steps of energy minimization using the limited memory Broyden–Fletcher–Goldfarb–Shanno method. Key mutations for Arg^204^ and His^269^ identified in SSM were then subjected for a physically more rigorous assessment for the change in free energy of mutation using the MD-based MM-PBSA method.

### Cell culture and imaging

HEK293 cells were cultured and maintained at 37 °C in a 5% CO_2_ humidified incubator supplemented with Dulbecco’s modified Eagle’s medium (low glucose) (Thermo Fisher Scientific) containing 10% fetal bovine serum (Nucleus Biologics) and 1% Pen/Strep (Thermo Fisher Scientific). After trypsinization, cells were transferred to a 35 mm glass bottom dish (MatTek). Cells were then grown to 70 to 80% confluency and transfected with 2.5 μg DNA/dish using Lipofectamine 2000 (Invitrogen) according to the manufacturer’s protocol. Transfections were carried out for 24 h. Cells were stained with the Hoechst 33342 nuclear stain and the WGA-Alexa647 membrane stain (Thermo Fisher Scientific) and imaged using a Nikon A1R confocal microscope with a plan apochromat 60× 1.4 NA oil objective. The 488 nm laser line was used for the excitation of EGFP, the 405 nm line was used for the excitation of the Hoechst 33342 nuclear stain and the 640 nm line was used for the excitation of the WGA-Alexa647 PM stain.

To calculate PM localization, the images were opened in ImageJ software and the channels were split. For the green channel, the background was subtracted using a rolling ball radius of 50.0 and contrast enhanced using a pixel size of 0.3%. The threshold was adjusted to select the cell outer boundary, and the region was filled using the “Fill Holes” command. The Wand tool was used to select the region of interest (ROI) and moved to the ROI manager. Then, the boundary was eroded so that the area enclosed would represent the cytosol. Again, the Wand tool was used to select the ROI and moved to the ROI manager. The area between the two regions of interest represents the PM area and can be selected using the XOR function. The green fluorescence intensity was measured in the PM and cytosol. PM localization was calculated as follows: (PM intensity/(PM intensity + cytosol intensity))∗100. WT EGFP-VP40, and EGFP was used to normalize the PM localization signal to 100% and 0%, respectively, to assess several VP40 mutations for PM localization. At least 30 cells were imaged for WT, EGFP, and each mutation over three independent experiments.

### Lipids and lipid binding assay

POPC (#850457), POPE (#850757), POPS (#840034), and PI(4,5)P_2_ (#850155) were purchased from Avanti Research, A Croda brand and stored in chloroform (or chloroform/methanol/dH_2_0 for PI(4,5)P_2_) at −20 °C until use. For MLV preparation, lipid mixtures were prepared as previously described (22) at the indicated lipid compositions and dried down to lipid films under a continuous stream of N_2_. For MLV binding experiments, final lipid concentrations were 1 mM and final protein concentrations 5 μM in each sample. Equal volumes of supernatant or pellet were run using a 10% acrylamide gel *via* SDS-PAGE and Coomasie Brilliant Blue G-250 staining was performed to detect protein bands for quantification using densitometry analysis in ImageJ. The protein bound fraction was determined using density_pellet_/(density_supernatant_ + density_pellet_).

### VLP production and purification

HEK293 cells were grown in a 100 mm round dish and transfected with 10 μg of DNA as previously described (28). VLPs were collected 48 h post transfection and budding assays were performed as described previously (28). In brief, VLP containing supernatants were harvested from cells and clarified through low-speed centrifugation (1000*g* for 10 min). Clarified VLPs were loaded onto a 20% sucrose cushion in STE buffer (10 mM TRIS, pH 7.6, containing 100 mM NaCl and 1 mM EDTA), isolated through ultracentrifugation (110,000*g* for 3 h), and resuspended in STE buffer. Cell lysate samples were harvested and lysed on ice with RIPA buffer (50 mM Tris, pH 7.4, containing 150 mM NaCl, 5 mM EDTA pH 8, 1% Triton X-100, 0.1% SDS, and 0.5% deoxycholic acid) supplemented with 1% Halt Protease Inhibitor Cocktail (Thermo Fisher Scientific). Samples were stored at −20 °C for Western blot analysis.

### Western blot analysis

Proteins from cell lysates and VLPs were separated by size using 10% SDS-PAGE. Following transfer onto a nitrocellulose membrane, the membranes were blocked with 5% milk in TBS-T (20 mM Tris, pH 8.0, containing 150 mM NaCl and 0.1% Tween) and analyzed with their respective antibodies. GFP-VP40 was detected using the anti-GFP HRP antibody (Abcam). GAPDH was detected using the mouse anti-GAPDH primary antibody (MilliporeSigma) and the sheep anti-mouse-HRP secondary antibody (Abcam). Antibodies were detected using an enhanced chemiluminescence detection reagent (Bio-Rad) and imaged on an Amersham Imager 600. All quantitative analysis were performed using densitometry analysis in ImageJ. Following enhanced chemiluminescence detection, VP40 cell lysate (VP40_CL_) expression was normalized to the respective GAPDH band density. The relative budding index was calculated according to the ratio of density_VLP_/density_CL + VLP_ (where density_VLP_ is the VP40 VLP band density and density_CL + VLP_ is the VP40 cell lysate + VLP band density).

### MD simulations

Starting with the homology model of WT VP40 (PDB 4LDB) and the most favorable models of R204H and H269R mutants generated by the flex_ddG protocol of Rosetta (45, 46), protein preparation was performed with the Protein Preparation Wizard in Schrödinger suite 2019 (47). This involves addition of missing hydrogen atoms to the heavy atoms, followed by optimization of their positions by a restrained minimization of the protein structure using the OPLS3e force field parameters and a RMSD cutoff of 0.30 Å to enable appropriate termination of the minimization (48, 49). The final H-bond optimization for the polar amino acids was done using PROPKA rules over a wide pH range of 5.0 to 9.0. The systems were then prepared according to a standard protocol with the protein being modeled with the ff14SB all-atom AMBER force field (50). The conformational sampling and energetics evaluation were enabled by CUDA accelerated pmemd module of AMBER 22 (51, 52). Starting with the homology model of VP40 and the mutants, explicit solvation in an octahedral TIP3P water box extending 12.0 Å from the protein in all directions was performed. Na+ and Cl−counterions were added to models a 0.15 M salt concentration in LEaP. The particle mesh Ewald method was used to model long-range electrostatic interactions and periodic boundary conditions with a 10 Å cutoff for nonbonded van der Waals interactions. The hydrogen atoms were constrained to the equilibrium positions of the heavy atoms using the SHAKE algorithm while a 2 fs time step was used for numerical integration. Initial minimization of the protein was carried out in increments of 1000 steps by gradually decreasing harmonic restraints of the protein heavy atoms. The system was then heated from 0K to a target temperature of 300K over 30 ps under NVT conditions followed by an NPT equilibration at 300K for 100 ps. The temperature and pressure were maintained using a Langevin thermostat and a Berendsen barostat with isotropic scaling, respectively.

Using the last frame of the equilibration protocol, conformational sampling and energetics evaluation of the systems were performed for 100 ns in triplicate to yield a total of 300 ns for each of the systems—WT VP40, R204H, and H269R mutants. Each replicate had identical starting coordinates but randomized initial velocities that ensured independent and unbiased sampling of protein conformations and energetics. Post simulation analyses were performed with cpptraj module of AmberTools to select appropriate snapshots for MM-PBSA and calculation of SASA analyses (53). The SASA measurements for Arg^204^, His^269^ and their mutants were carried out using the linear combinations of pairwise overlaps (LCPO) method (54). Plotting of the time series data was achieved with ggplot2 and R (55, 56). The resultant values for change in relative free energy of mutation and SASA have been averaged over the three individual replicate simulations and are expressed as mean ± SD.

The membrane association of EBOV VP40 dimer mutations was investigated through MD simulations. Three distinct systems were constructed using the CHARMM-GUI web server (57, 58): a WT system and two single-point mutation variants, H269R and R204H. The CHARMM-GUI mutation tool facilitated the introduction of these mutations. Membrane-protein complexes were prepared by aligning the VP40 dimer against a CHARMM-GUI built membrane for each system. MD simulations were performed using NAMD 3.0 (59) with the CHARMM36m force field (60). The lipid composition was complex and asymmetric, with CHOL:SM:PS:PIP_2_:PE:PC in the ratio 17:31:13:7:24:8 for the outer leaflet and 22:9:16:9:32:12 for the inner leaflet, similar to the composition used in our previous work (61). Each system underwent a six-step equilibration protocol followed by a 1 μs production run. Temperature and pressure were controlled using Langevin dynamics with a friction coefficient of 1 ps^−1^, and pressure was maintained using the Nose-Hoover Langevin piston (62). Long-range electrostatic interactions were treated using the particle mesh Ewald method (63). All hydrogen-containing covalent bonds were constrained using the SHAKE algorithm (64).

### MM-PBSA calculations

MM-GB/PBSA is a well-established MD-based method for estimation of binding free energies that was used to estimate the binding free energy (43). Starting with WT VP40 (PDB 4LDB), R204H, and H269R mutants from site saturation mutagenesis were simulated for 100 ns in triplicate under constant pressure and temperature (NPT) conditions. Post simulation analyses revealed low RMSD drifts throughout the duration of the individual simulations and hence the cumulative 300 ns simulations were used to estimate the changes in relative free energy of mutations using the MM-PBSA method. The calculations did not involve entropy calculations because of the large errors involved in the estimation of the entropic contribution toward the free energy as well as the overall low accuracy of entropy estimation.

## Data availability

All data are located in the manuscript and supporting information.

## Supporting information

This article contains supporting information.

## Conflict of interest

The authors declare that they have no conflicts of interest with the contents of this article.
