# Supplementary material for: Variants of the Ebola virus matrix protein VP40 have differential effects on oligomerization and plasma membrane interactions
Source: J Biol Chem. 2025 Jul 16;301(9):110489. doi: 10.1016/j.jbc.2025.110489 (PMC12409429; doi:10.1016/j.jbc.2025.110489)
Supplement: Supporting information [file mmc1.docx]

**Supporting Information**

Variants of the Ebola virus matrix protein VP40 have differential effects on oligomerization and plasma membrane interactions

Balindile B. Motsa^1^, Barsha Bhowal^1^, Yogesh B. Narkhede^2^, Ukesh Karki^3^, Valentina Toro Ramirez^1,4^, Samuel W. Eger^1^, Olaf Wiest^2^, Prem P. Chapagain^3,5^, and Robert V. Stahelin^1*^

^1^Borch Department of Medicinal Chemistry and Molecular Pharmacology and the Purdue Institute of Inflammation, Immunology, and Infectious Disease, Purdue University, West Lafayette, IN 47907, USA.

^2^Department of Chemistry and Biochemistry, University of Notre Dame, Notre Dame, IN 46556, USA.

^3^Department of Physics, Florida International University, Miami, FL 33199, USA.

^4^Pharmaceutical Chemistry, Universidad CES, Medellin, Colombia

^5^Biomolecular Sciences Institute, Florida International University, Miami, FL 33199, USA.

*To whom correspondence should be addressed: Robert V. Stahelin, Borch Department of Medicinal Chemistry and Molecular Pharmacology, Purdue University, 207 S. Martin Jischke Drive, West Lafayette, IN 47907, USA. TEL: 1-765-494-4152; Email: [rstaheli@purdue.edu](mailto:rstaheli@purdue.edu)

Short title: VP40 patient mutation alters lipid binding

Keywords: Ebola virus, electrostatics, lipid-protein interactions, phosphatidylinositol-4,5-bisphosphate, phosphatidylserine, plasma membrane, virus assembly, virus budding, VP40

**Supplementary Figure 1.** **Site saturation mutagenesis for Arg^204^ of wild type VP40**. The reported values and error bars have been calculated over 100 models that represent 100 cycles of mutagenesis. Positive values of predicted ΔG imply destabilization of dimer while negative ΔG values imply increased stabilization of the dimer. The Arg204Pro mutation clearly stands out as destabilizing for the dimer when compared to other 18 mutations.


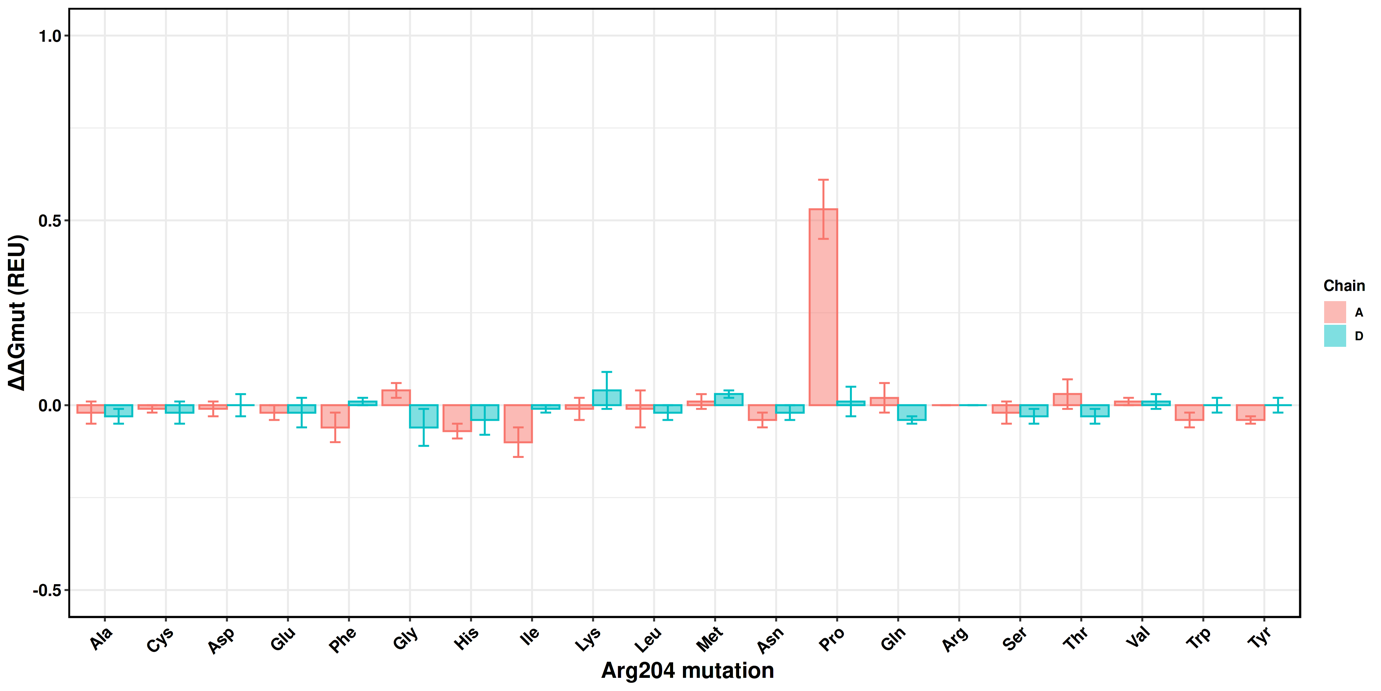


**Supplementary Figure 2.** **Solvent accessible surface area (SASA) calculations for individual simulations of Arg^204^ and R204H mutants**. The dashed line and the associated number depicts the SASA for Arg^204^ in PDB 4LDB as calculated using a probe of radius 1.4 Å. The R204H mutation results in noticeable upward shifts in SASA irrespective of the chain where the mutation was carried out or the simulation. The plots on the left indicate SASA drifts for wild type EBOV VP40, while the ones on the right indicate Arg204His mutants.

**
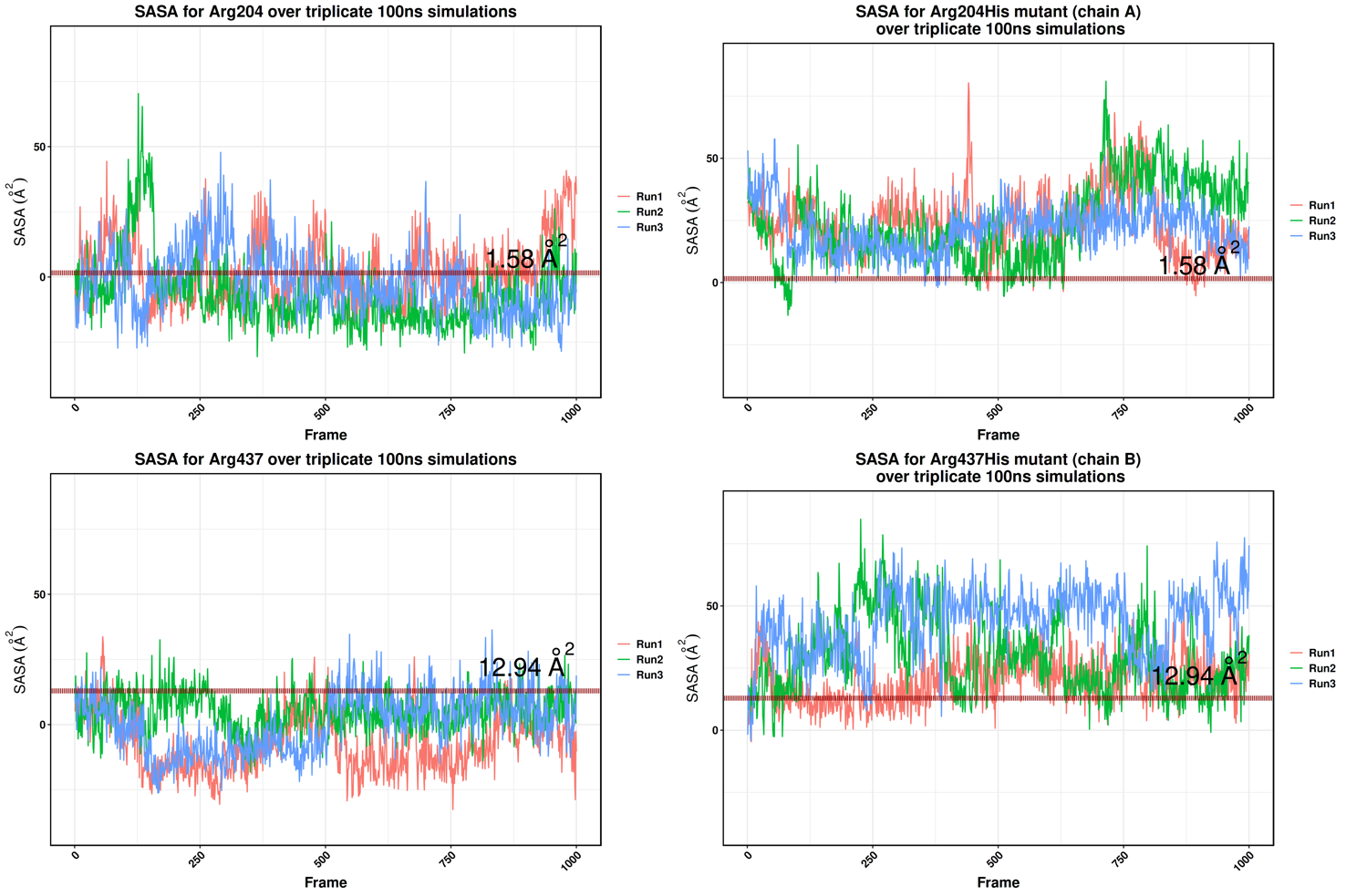
**

**Supplementary Figure 3.** **Solvent accessible surface area (SASA) calculations for individual simulations of His^269^ and H269R mutants**. The dashed line and the associated number depicts the SASA for His^269^ in PDB 4LDB as calculated using a probe of radius 1.4 Å. The H269R mutation results in noticeable upward shifts in SASA irrespective of the chain where the mutation was carried out or the simulation. The plots on the left indicate SASA drifts for wild type EBOV VP40, while the ones on the right indicate His269Arg mutants.

**
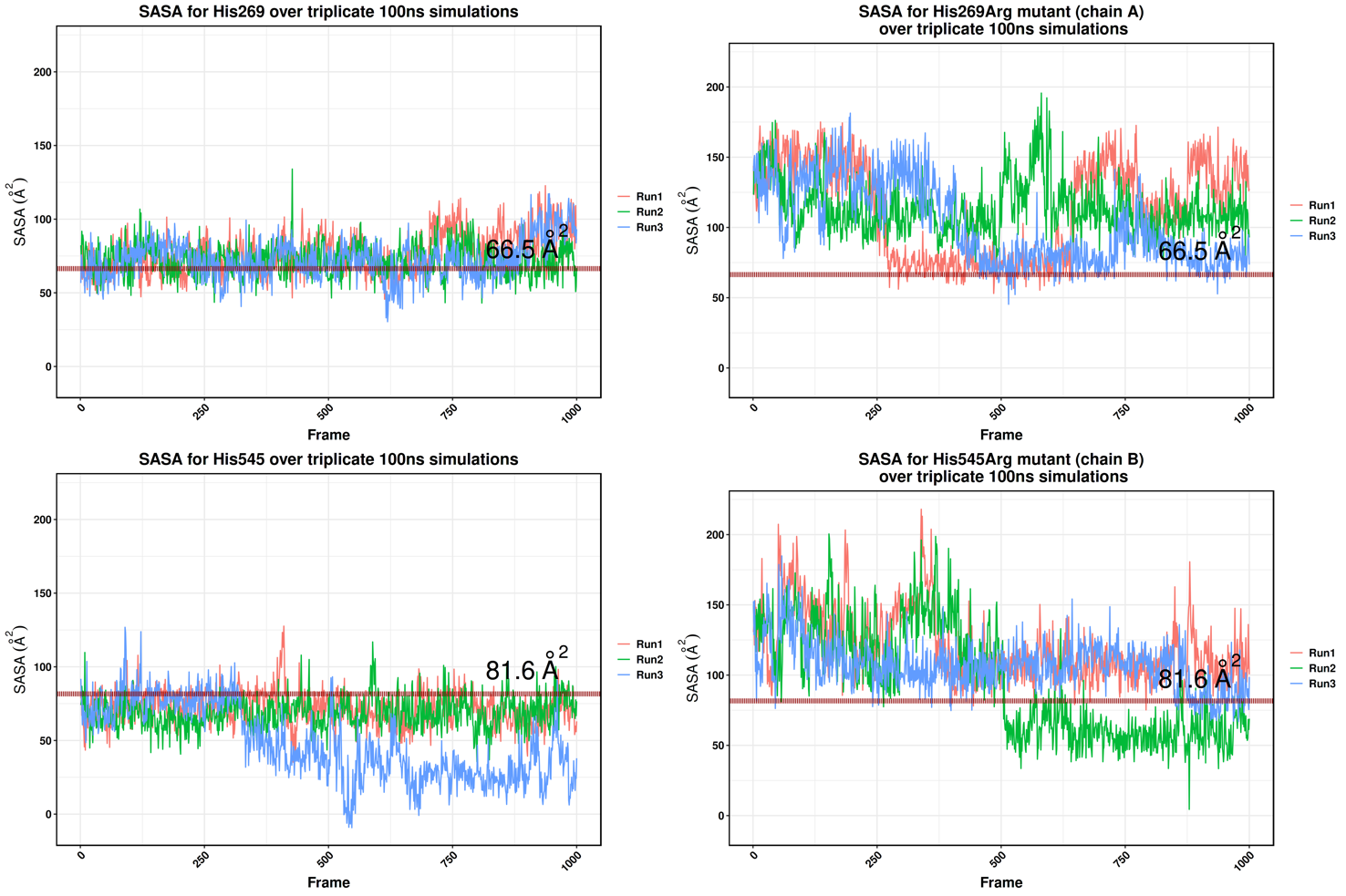
**

**Supplementary Figure 4.** **Root mean square deviation (RMSD) calculations**. *A*, Heavy atom RMSD for Arg^204^ and Arg^437^ in wild type VP40, *B*, Heavy atom RMSD for His^269^ and His^503^ in wild type VP40; *C*, Heavy atom RMSD for Arg204His and Arg437His mutants; *D*, Heavy atom RMSD for H269R and H503R mutants.


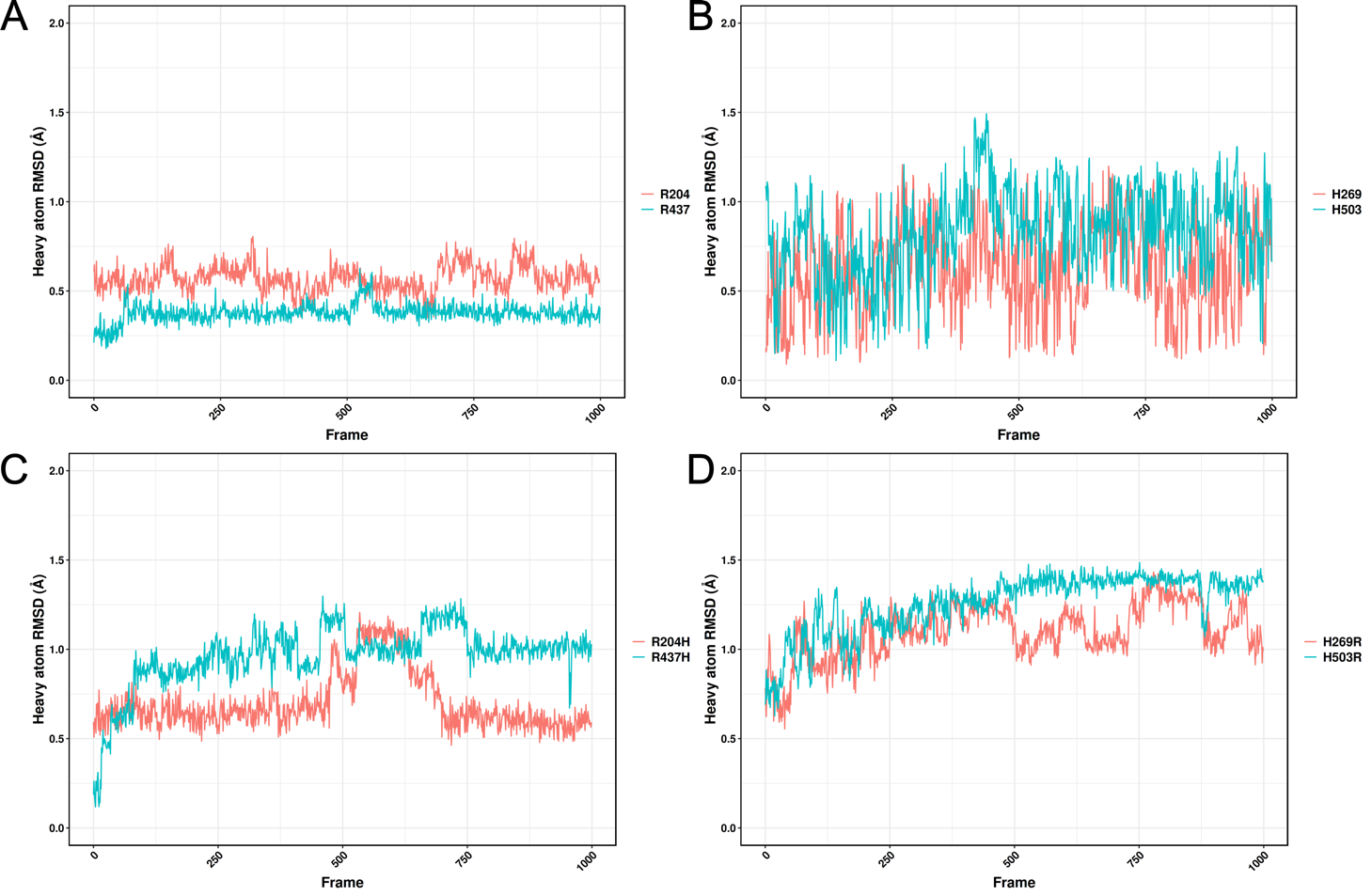


Table S1: **Relative binding free energy calculations for wild type VP40, chain A R204H and chain B R204H mutants.** Each value has been calculated over an ensemble of 1000 frames corresponding to 100 ns of simulation under constant pressure and temperature. The units for change in free energy are kcal/mol and SD is standard deviation.

| System | ΔG-r1 | SD | ΔG-r2 | SD | ΔG-r3 | SD | Average ΔG | SD | Difference |
| --- | --- | --- | --- | --- | --- | --- | --- | --- | --- |
| VP40 | -21.3 | 6.5 | -20.9 | 6.3 | -17.7 | 8.4 | -20.0 | 7.1 | 0.0 |
| Chain A mutant | -20.3 | 7.0 | -18.4 | 7.2 | -13.2 | 6.9 | -17.3 | 7.1 | 2.7 |
| Chain B mutant | -18.8 | 6.8 | -19.7 | 7.1 | -20.0 | 7.3 | -19.4 | 7.1 | 0.6 |

Table S2: **Relative binding free energy calculations for wild type VP40, chain A H269R and chain B H269R mutants.** Each value has been calculated over an ensemble of 1000 frames corresponding to 100 ns of simulation under constant pressure and temperature. The units for change in free energy are kcal/mol and SD is standard deviation.

| System | ΔG-r1 | SD | ΔG-r2 | SD | ΔG-r3 | SD | Average ΔG | SD | Difference |
| --- | --- | --- | --- | --- | --- | --- | --- | --- | --- |
| VP40 | -21.5 | 6.7 | -20.2 | 6.8 | -18.8 | 6.7 | -20.5 | 4.0 | 0.0 |
| Chain A mutant | -18.3 | 7.4 | -17.8 | 8.3 | -19.6 | 7.4 | -19.4 | 4.2 | 1.1 |
| Chain B mutant | -21.7 | 6.6 | -20.3 | 6.7 | -17.9 | 8.4 | -18.8 | 4.4 | 1.7 |
